# Prevalence and Genotype Distribution of High-Risk Human Papillomavirus Infection Among Sub-Saharan African Women: A Systematic Review and Meta-Analysis

**DOI:** 10.3389/fpubh.2022.890880

**Published:** 2022-07-08

**Authors:** Ayichew Seyoum, Nega Assefa, Tadesse Gure, Berhanu Seyoum, Andargachew Mulu, Adane Mihret

**Affiliations:** ^1^College of Health and Medical Sciences, Haramaya University, Harar, Ethiopia; ^2^Armauer Hansen Research Institute, Addis Ababa, Ethiopia

**Keywords:** prevalence, genotypes, high-risk, HPV, sub-Saharan, infection

## Abstract

**Background:**

Among sub-Saharan African women, cervical cancer is steadily increasing with more than 75,000 new cases and 50,000 deaths annually. Due to the vast ethno geography variation, Africa harbors heterogeneous genotypes of HPV. High-risk HPV [hr HPV] genotypes such as hr HPV-16,−18,-35, and−52 are abundantly reported in sub-Saharan Africa. The purpose of this systematic review and meta-analysis is to generate an evidence on the prevalence and the genotype distribution of hr HPV among sub-Saharan African countries.

**Methods:**

The review was conducted by following the preferred reporting items for systematic reviews and Meta-analysis. PubMed/Medline, Embase, Scopus, Google Scholar, Heath Technology assessment and Cochrane Library databases were used to retrieve published original studies between 2001 and 2021. It included studies that used PCR-based or hybrid testing to assess the presence of HPV DNA in a cervical biopsy, cervical swelling, and vaginal swelling. Statistical software for data science (STATA V16) software using a random-effects model was used to determine the pooled prevalence and type-specific distribution of HPV with 95% confidence intervals (CI). The I-squared statistic was used to describe the level of heterogeneity. The study protocol is registered on PROSPERO with reference number CRD42022311157.

**Results:**

The review included 27 studies conducted in 19 sub-Saharan countries. A total of 16,506 study participants from 27 studies were included in a systematic review and 5,303 of them were infected with the hr HPV infection. Out these, only 3,075 of them were eligible for meta-analysis. The incidence proportion of estimatesof hr HPV infection among study participants with different health conditions ranges from 10.7 to 97.2% while the pooled incidence proportion of estimates is 34% (95%CI: 29–39). Among 3,075 women, 424 (13.8%), 305 (9.9%) and 279 (9%) were infected with HPV-16,−52 and−18, respectively. HPV-16 and−52 are the main genotypes causing the hr HPV infection in the Eastern and Southern African sub-contents, whereas HPV-16 and−35 are the main genotypes in the Western African countries.

**Conclusions:**

Depending on several factors, especially women's health conditions, the high rate of hr HPV infection with inconsistent genotype distribution shows that it is a growing public health challenge in sub-Saharan African countries. Therefore, to implement a vaccination-based prevention strategy and be effective, considering factors associated with hr HPV infection is crucial.

## Introduction

Human papillomavirus (HPV) infection is a sexually transmittable disease caused by Human papillomavirus (1). The virus is divided into five genera based on differences in their nucleotide sequence: alpha, beta, gamma, delta, and mue (2). The mucosal/genital HPV are part of the alpha papillomavirus (alpha-PV) and based on their involvement in malignant lesions, some are oncogenic (high-risk) and others are non-oncogenic (low-risk). Thus, 15 types of HPVs (HPV-16,−18,−31,−33,−35,−39,−45,−51,−52,−56,−58,−59,−68,−73, and−82) are classified as high-risk (hr) HPV which are responsible for dysplasia and cancer. The other 12 types of HPVs (HPV-6,−11,−40,−42,−43,−44,−54, - 61, - 70,−72,−81 and CP 6108) are low-risk types often cause low-grade mild dysplasia, genital warts and respiratory papillomatosis. The rest three HPVs (HPV-26,−53, and−66) are classified as probable high-risk types (3).

Cervical cancer (CC) is a malignant neoplasm arising from cells originating in the cervix. It is usually a slow-growing cancer that may not have clear symptoms. Consistent with research, 99% of CC cases are directly related to the hr HPV genotypes, and about 70% of them are caused by HPV-16 and HPV-18, worldwide (4). But this reality is changing in women-living-with-HIV (WLWH). That is, HPV-58 has been proven to be the second leading cause of cervical cancer after HPV-16 (5).

CC caused by hr HPV covers 5.2% of all cancers worldwide. This means that out of the total cancer patients in high income and low income countries, 2.2 and 7.7% are CC patients, respectively (6, 7). In sub-Saharan Africa countries, the prevalence of hr HPV infection ranges from 10.7% in Ghana (8) to 90.8% in Cote d' Ivorie (9). However, the prevalence of hr HPV infection among WLWH can reach up to 97% (10). Similar to the prevalence, the genotypes distribution of hr HPV also varies depending on the vicinity of the study and the health status of women participated in the study.

Studies in the southern African countries have shown that the hr HPV genotypes that mainly cause CC vary from country to country. For example, HPV-16 and−52 in South Africa and Tanzania (11), HPV-16 and−35 in Zimbabwe (12), and HPV-53 and−68 in Madagascar (13) were the most common genotypes cause CC among women. Similarly, studies in east African countries have also shown that there is no uniform distribution of hr HPV genotypes. In Uganda, HPV-52 and−58 (14); in Tanzania, HPV-52 and−16 (15); in Kenya, HPV-58 and−16 (16); and in Ethiopia, HPV-16 and−52 (17) genotypes were identified as the main causes for hr HPV infection. The distribution of hr HPV genotypes in the West African countries has a different pattern from the southern and eastern African countries. In Senegal, HPV-52 and−31 (18), in Burkina Faso, HPV-52 and−59 (19), and in Nigeria, HPV-35 and−16 (20) genotypes were identified as the leading causes of the disease.

Although hr HPV infection is a global public health problem, there is a genotype-based effective vaccine (21). There are three licensed vaccines: Cervarix (Virus-Like Particles (VLPs) of late 1 (L1) from genotypes HPV-16 and−18); Gardasil (VLPs of L1 of HPV-6,−11,−16 and−18 genotypes) and Gardasil 9 (VLPs of L1 of HPV-6,−11,−16,−18,−31,−33,−45,−52 and−58 genotypes), and the WHO recommendation is that two doses of the divalent and tetravalent vaccines be applied. Since vaccination based on L1 VLPs only specifically protects against the included genotypes, trying to develop multivalent vaccines for all oncogenic genotypes is not possible, and it does not have therapeutic potential. This has generated interest in developing L2-based proteins, taking advantage of epitopes conserved between different genotypes. Although it has been seen that it does not generate immunity similar to L1, it has therapeutic potential by including early expression proteins. It also provides the possibility of including genotypes from other species that have been associated with skin cancer.

This means that to buy a vaccine and ensure its effectiveness, having valid evidence regarding the genotype distribution of the virus in a given locality is critical. Therefore, this systematic review and meta-analysis have prepared to fill the current information gaps regarding the prevalence and genotype distribution of hr HPV infection in sub-Saharan countries.

## Methods

The review protocol was prepared according to a statement recommendation made by the Preferred Reporting Items for Systematic Review and Meta-Analysis Protocols (PRISMA-P) in 2015 (Supplementary Table 1) (22). Besides, during the process of study selection, we strictly followed the National Institute of Health (NIH) Quality Assessment Tool for Observational Cohort and Cross-Sectional Studies guideline to assess the methodological quality of studies (Supplementary Table 2) (23). The International Prospective Register of Systematic Reviews (PROSPERO) has registered the protocol with reference number CRD42022311157.

### Search Strategy

We identified publications by systematic searches of PubMed/Medline, Embase, Scopus, Google Scholar, Heath Technology assessment and Cochrane Library, from August 1 to September 15, 2021. The identified records were downloaded with an appropriate format and linked to the Endnote. The Boolean operators “AND” and “OR” were used to link keywords/terms and fetch publications from PubMed / MEDLINE (NCBI) databases. The Medical subject heading (MeSH) terms that we used are indicated in Appendix 1. In addition, google scholar was used as a web-based source to retrieve studies that did not index on PubMed.

### Eligibility Criteria

We applied several inclusion and exclusion criteria that were defined a priori to the records identified. Publications eligible for inclusion were those studies that reported the prevalence of hr HPV infection among sub-Saharan African countries as a primary outcome. We used the following criteria to include the texts in this analysis. These are: If the full article and its related data are accessible: If at least 25 women participated in the study; If cervical histology results are confirmed by exfoliated cervical cells or fixed/fresh biopsy results; If the hr HPV prevalence is calculated and at least five genotypes identified. In addition to that, we included studies that used either the amplified polymerase chain reaction (PCR) or non-amplified genotyping methods as a method of detection. There were no restrictions on PCR primers' utilization. We also included studies conducted on the HPV DNA tissue sources including fixed or fresh biopsies and/or exfoliated cells. Similarly, we excluded abstracts with unrelated data, non-English language publications, and published without original data (reviews, correspondence, guidelines, letters, and editorials). We also excluded studies that reported results from a case series or case report, and qualitative data.

### Study Selection Procedure

**Figure 1:**
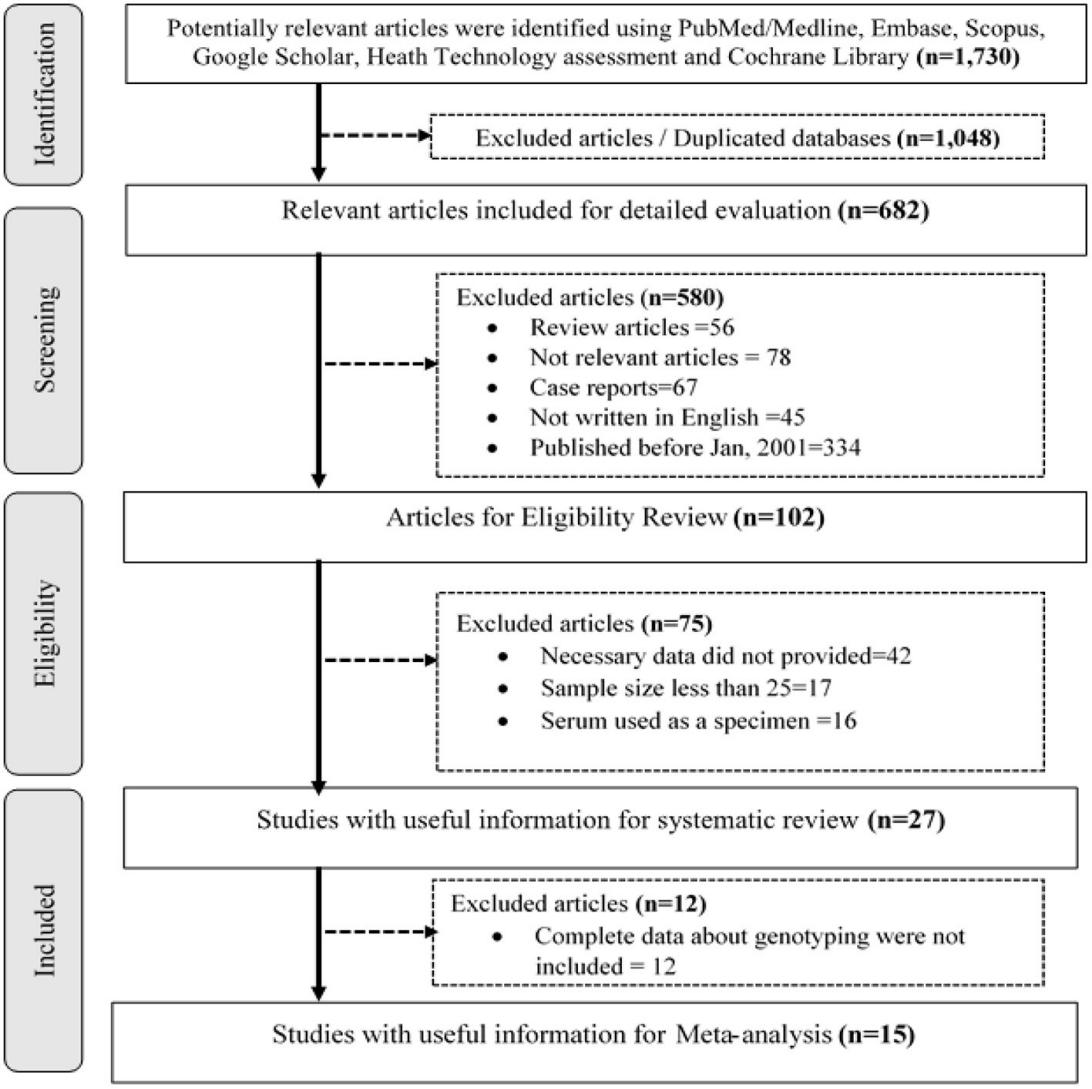
A flowchart shows the step-by-step selection procedure of publications.

First of all, we identified relevant articles. Then, we removed duplicated publications using Endnote, version x7 (Thomson Reuters, Stamford, CT, USA), and manual screening. The first two authors (AS and NA) independently appraised the titles and abstracts of the retained publications and selected the relevant articles for possible inclusion in the review. Accordingly, studies conducted to determine the prevalence of hr HPV and genotype distribution were kept for review and further analysis. Correspondingly, other authors (TG, BS, AM, and AM) reviewed the full text to assess the methodological quality of studies using the National Institute of Health (NIH) Quality Assessment Tool for Observational Cohort and Cross-Sectional Studies guideline (23). Finally, all authors thoroughly discussed and agreed to include articles that scored greater than or equal to the average point (7). As a result, 27 and 15 published articles were selected for systematic review and meta-analysis, respectively (Figure 1).

### Methodological Quality Assessment

The NIH Quality Assessment Tool for Observational Cohort and Cross-Sectional Studies checklist was used to evaluate the methodological quality of the retained publications. Accordingly, all studies that fulfilled at least 50% of the quality requirement as per the average positive score of the appraisers were considered for this review (Supplementary Table 2).

### Data Extraction

We used the data abstraction format prepared in a Microsoft excel spreadsheet to extract all the necessary information for systematic review and meta-analysis. Two non-blinded investigators (AS and NA) extracted the data independently, reviewed it for discrepancies, and finally reached a consensus through discussion. The following variables were extracted: study design, year of publication, sample size, continental category of the study (according to WHO classification), study location, detection methods, clinical sample type, prevalence of hr HPV and type specific prevalence (genotypes) (Supplementary Table 3).

### Statistical Analysis

Statistical pooling for incidence proportion of estimates was performed according to the random-effects model using a Statistical software for data science (STATA V16). The random-effects model of analysis was assumed since the studies identified were observational in nature and they had both clinical and methodological variabilities.

**Figure 2:**
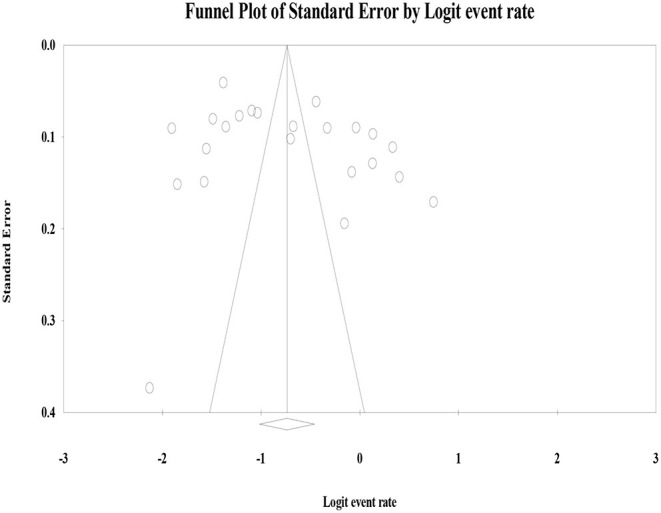
Funnel plot of standard error by effect size for publication bias.

The heterogeneity of studies was evaluated based on the Cochrane *Q* and *I*^2^ tests as well as Q/df (degree of freedom) ratio results. Thus, the Cochrane Q test (*p* = 0.1), Q/df = 1, and *I*^2^ = 50% were considered as cutoff points to mark heterogeneity and to select the effective model of analysis. The forest plots were employed to present the pooled prevalence of hr HPV infection and genotypes. In line with this, subgroup analyses were carried out to explain patient features in sub-groups with the potential to account for the differences in the effect sizes of the hr HPV genotypes. Publication bias (or small-study effects) was assessed by a graphical inspection of funnel plot (Figure 2). Next, Egger's regression and Begg's correlation tests were performed to test the presence of publication bias.

### Ethical Statement

For this study, ethical approval from the Institutional Ethics Review Board is not required.

## Results

### Selection of Studies for Review

A total of 1,730 research citations that met the requirements of the National Institute of Health (NIH) Quality Assessment Tool for Observational Cohort and Cross-Sectional Studies guideline were retrieved (Figure 1).

For systematic review, 27 studies from 19 sub-Saharan African countries, totaling 16,506 study participants were included. Out of these, 5,303 were infected with hr HPV genotypes. Similarly, among the selected 27 studies for systematic review, only 15 studies involving 7,924 study participants had complete information on the genotype distribution of HPV. Of these, 2,188 women were from East Africa and 1,660 were from southern Africa. The remaining 4,076 women were from the West African countries (Table 1).

**Table 1:** A summary table of original articles retrieved for systematic review and meta-analysis from the search strategy, 2021.

**References**	**Country**	**Tested, *N***	**HPV +ve, *n***	**Event rate (%)**	**95% CI**
**Studies from East Africa**					
Teka et al. (24)^†^	Ethiopia	764	157	20.5	20.8–20.2
Ali et al. (1)^†^	Ethiopia	366	50	13.7	10.5–17.6
Asiimwe et al. (2)^†^	Uganda	314	54	17.2	13.4–21.8
Blossom et al. (14)^†*^	Uganda	106	49	46.2	37.0–55.7
Dartell et al. (15)^†^	Tanzania	3,603	725	20.1	18.8–21.5
De Vuyst et al. (16)^†*^	Kenya	496	208	41.9	37.7–46.3
Leyh-Bannurah et al. (17)^†*^	Ethiopia	537	94	17.5	14.5–21.0
Mayaud et al. (5)^†*^	Tanzania	561	190	33.9	30.1–37.9
Rahman et al. (25)^†*^	Kenya	488	240	49.2	44.8–53.6
Castle et al. (26)^†^	Ethiopia	1,022	257	25.2	21.2–29.4
**Studies from southern African countries**					
Rosa Catarino et al. (27)^†*^	Madagascar	1081	424	39.2	36.4–42.2
Dols et al. (11)^†^	South Africa	258	215	83.3	78.3–87.4
Gravitt et al. (28)^†*^	Zimbabwe	423	226	53.4	48.7–58.1
Sahasrabuddhe et al. (10)^†^	Zambia	145	141	97.2	92.9–99.0
Tayib et al. (29)^†*^	South Africa	156	106	67.9	60.2–74.8
**Studies from West Africa**					
ZoaAssoumou et al. (30)^†*^	Gabon	200	120	60.0	53.1–66.6
Domfeh et al. (8)^†^	Ghana	75	8	10.7	5.4–19.9
Jaquet et al. (9)^†^	Cote d' Ivorie	510	463	90.8	87.9–93.0
Kunckler et al. (31)^†*^	Cameroon	1012	187	18.5	16.2–21.0
Manga et al. (32)^†^	Nigeria	208	100	48.1	41.4–54.9
Mbaye et al. (18)^†*^	Senegal	936	214	22.9	20.3–25.7
Obiri-Yeboah et al. (33)^†*^	Ghana	329	192	58.4	53.0–63.6
Okolo et al. (20)^†*^	Nigeria	932	245	26.3	23.6–29.2
Ouedraogo et al. (34)^†^	Burkina Faso	256	230	90.0	85.0–93.0
Piras et al. (35)^†*^	Benin	427	142	33.3	28.9–37.9
Wall et al. (36)^†^	Gambia	1,061	138	13.0	11.1–15.2
Kuassi-Kpede et al. (37)^†*^	Togo	240	128	53.3	47.0–59.6
Total		16,506	5,303		

† *Selected for systematic review*,

* *selected for meta-analysis*,

†* *selected for both*.

### Prevalence of hr HPV Infection

Our systematic review reports hr HPV prevalence that varies by geographic location. Indeed, we also report that the prevalence and genotype differences have been seen among studies conducted in a country (38).

Among the sub-Saharan African women who visited the health facilities for gynecological problems, the lowest prevalence of hr HPV infection was 10.7% (95% CI: 5.4–19.9) (8). However, we found that the highest prevalence of infection varied depending on the health status of the study participants in the study. For example: 90.8% (95% CI: 87.9–93.0) among cervical cancer suspected women in Cote d' Ivorie (9), 94.1% (95% CI: 86.6–97.5) among cervical cancer patients in Sudan (39), and 97.2% (95% CI: 92.9–99) among WLWH in Zambia (10) (Table 1).

### Type Specific Prevalence of hr HPV Infection

**Figure 3:**
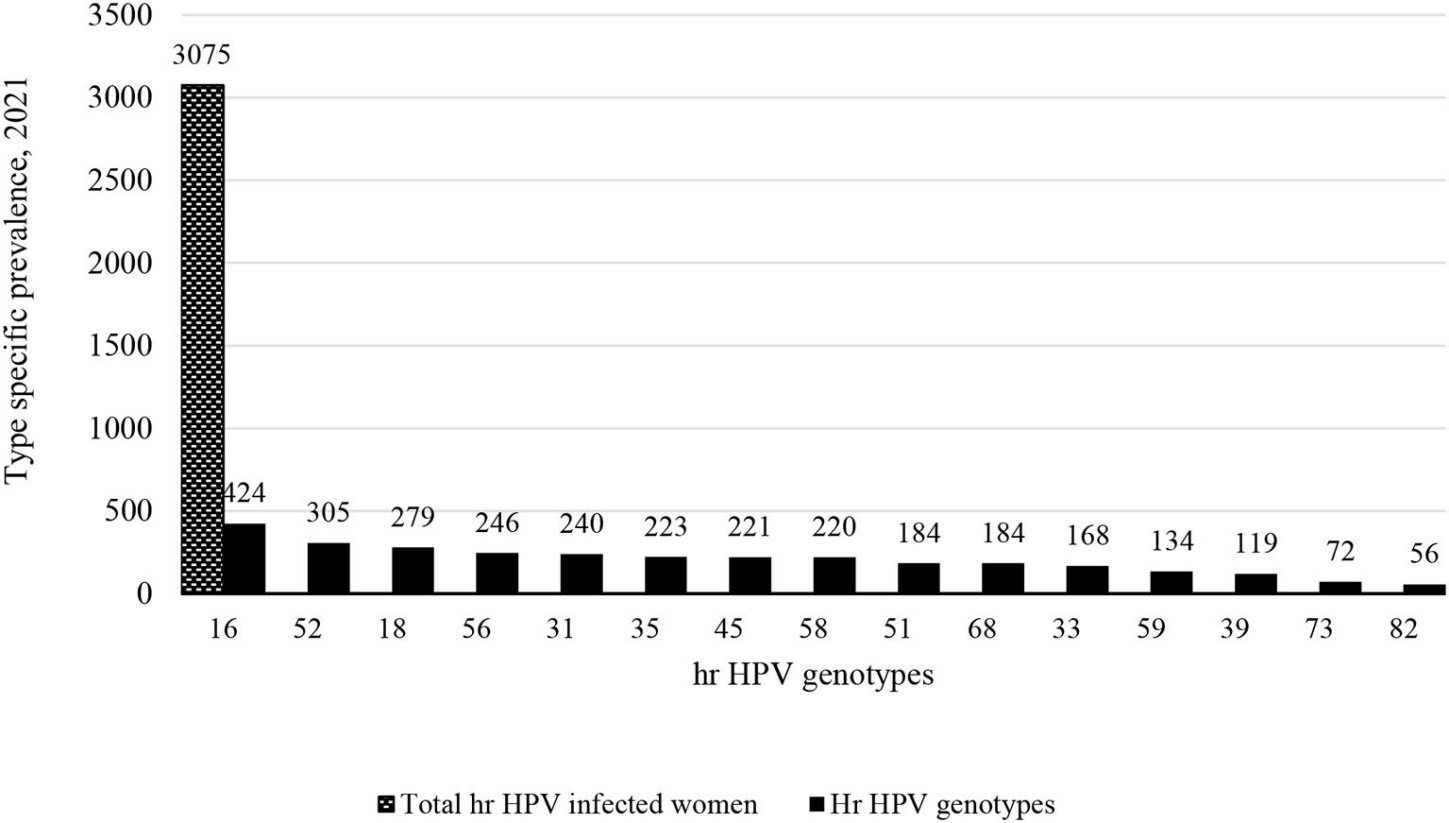
Type specific prevalence of hr HPV infection in sub Saharan African countries, 2021.

Out of 5,303 study participants infected with hr HPV, we included 3,075 women from 15 studies who met the inclusion criteria (data on more than 5 genotypes). Accordingly, 424 (13.8%) women were primarily infected by HPV-16. As well as, 305 (9.9%) and 279 (9%) women were infected by HPV-52 and HPV-18 (Figure 3). Full detail available on Supplementary Table 3.

### The Pooled Prevalence of hr HPV Infection From Meta-Analysis

**Figure 4:**
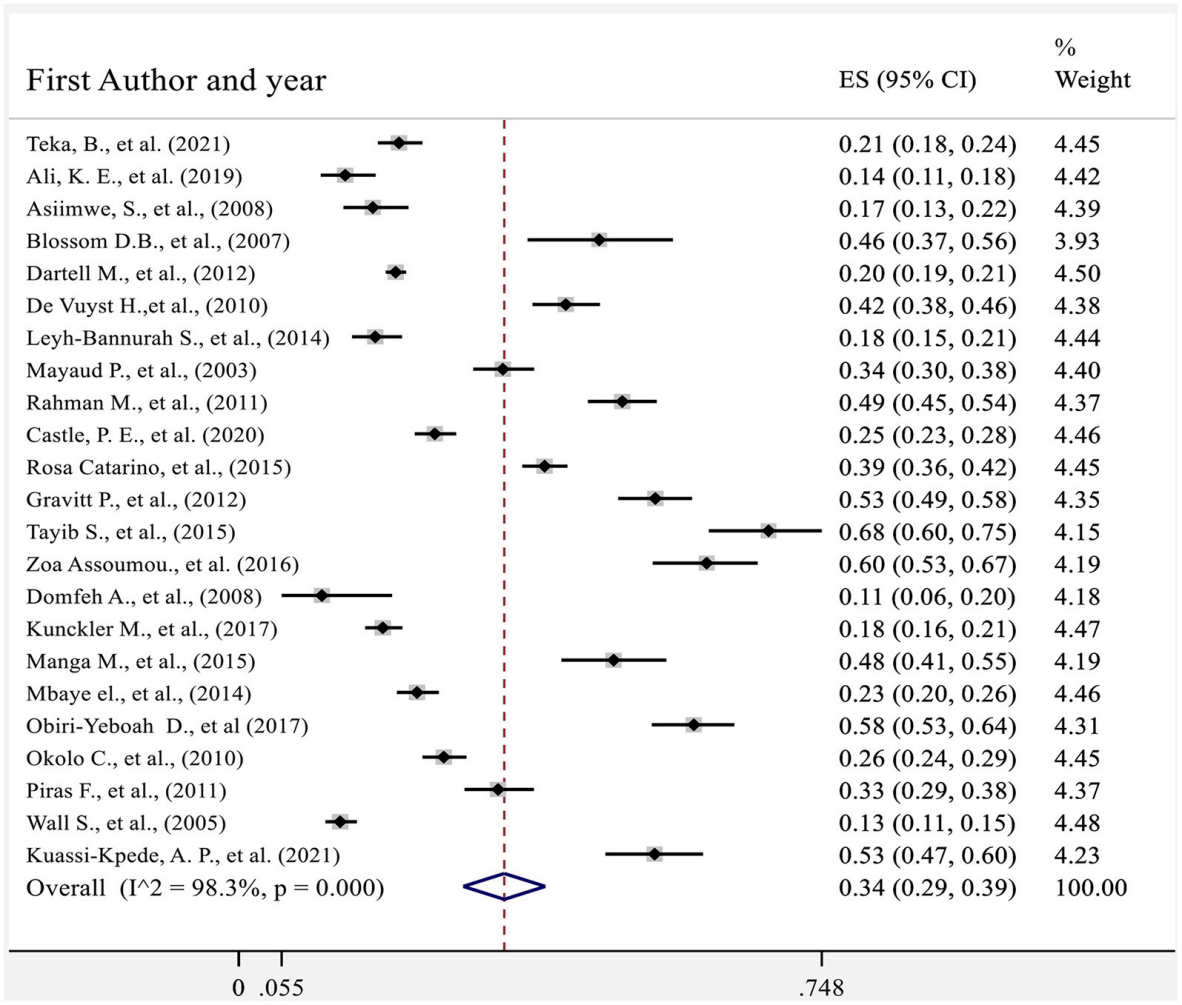
Forest plot showing the pooled proportion of hr HPV in sub-Saharan African countries 2021.

The collective analysis of 23 studies indicated that the pooled prevalence of hr HPV in sub-Saharan African countries was 34% (95%CI: 29–39) (Figure 4). The analysis also found that there was a significant heterogeneity between studies (*Q* = 1,180.2, *p* < 0.1, df = 22, *I*^2^ = 98.3%). The heterogeneity might be observed as a result of combining different studies that used different laboratory methods of diagnosis (Real-time PCR and Hybrid capture 2) and study participants who had different levels of health condition during the time of enrollment.

Graphical visualization of the funnel plot found its symmetrical appearance which gave us hint about absence of the publication bias (Figure 2). This visual inspection was further tested by using Egger's regression and it also showed no evidence of the small-study effects (effect estimate: 1.72; 95% CI: −1.36–14.35; *p* = 0.100). Additionally, Begg's correlation test revealed no evidence of the publication bias (*Z* = 0.92; *p* = 0.35).

### The Sub-group Meta-Analysis of the Prevalence of hr HPV

**Figure 5:**
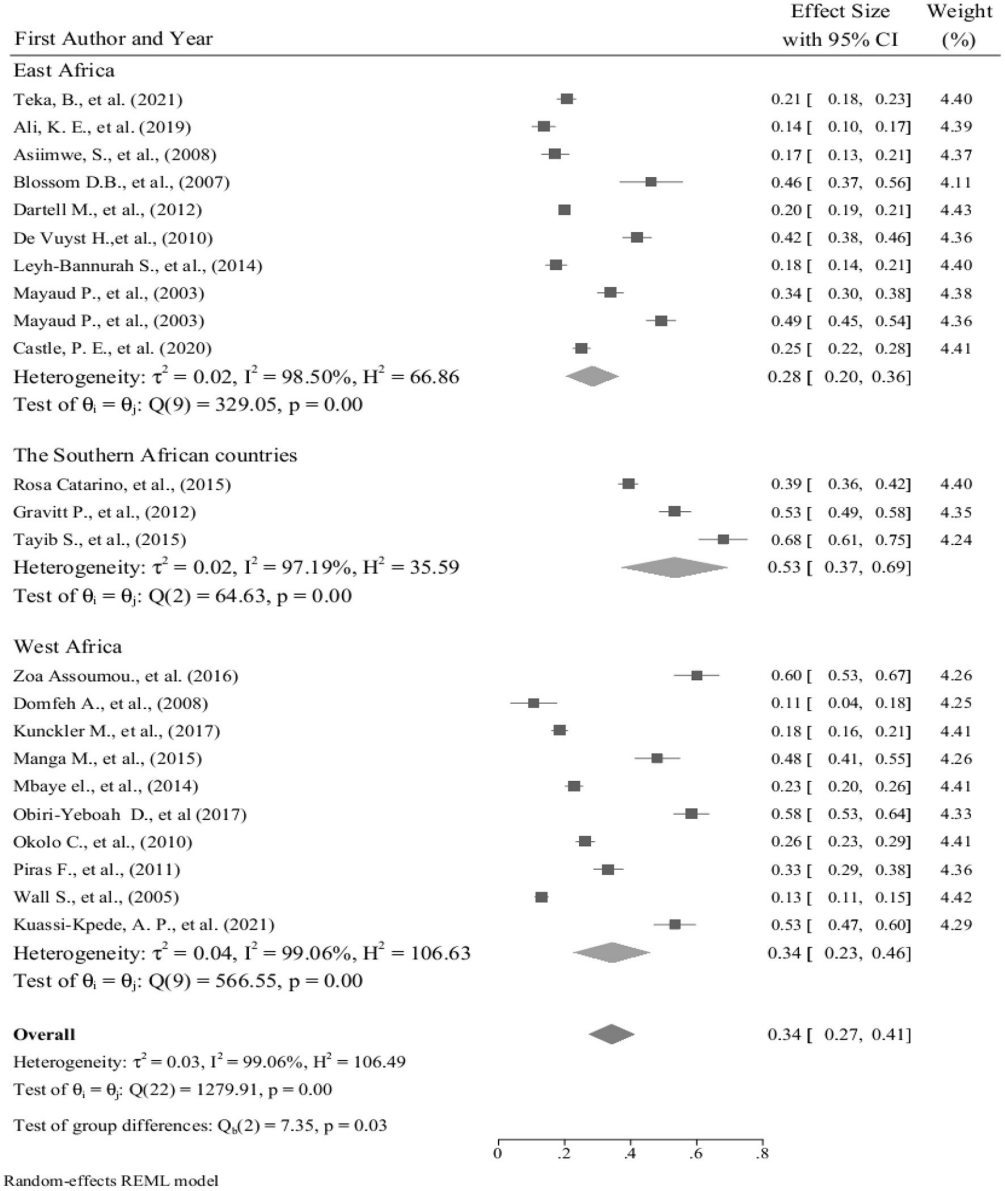
Forest plot showing sub-group analysis of the prevalence of hr HPV infection in sub-Saharan African countries, 2021.

We conducted the subgroup analysis of the prevalence of hr HPV infection by dividing the countries into East Africa, West Africa, and Southern Africa sub-groups. As compared to the total sub-group prevalence of the hr HPV infection 34% (95% CI: 23–46); the highest prevalence, 53% (95% CI: 37–69) was found among studies in Southern African countries. However, contrary to this, the lowest sub-group prevalence of hr HPV, 28% (95% CI: 20–36) was observed among the East African countries (Figure 5).

### Type-Specific Sub-group Prevalence of hr HPV From Meta-Analysis

The type-specific sub-group prevalence of hr HPV from meta-analysis was done by sub-grouping the sub-Saharan African countries into the Eastern, Southern, and Western sub-Saharan African regions.

**Figure 6:**
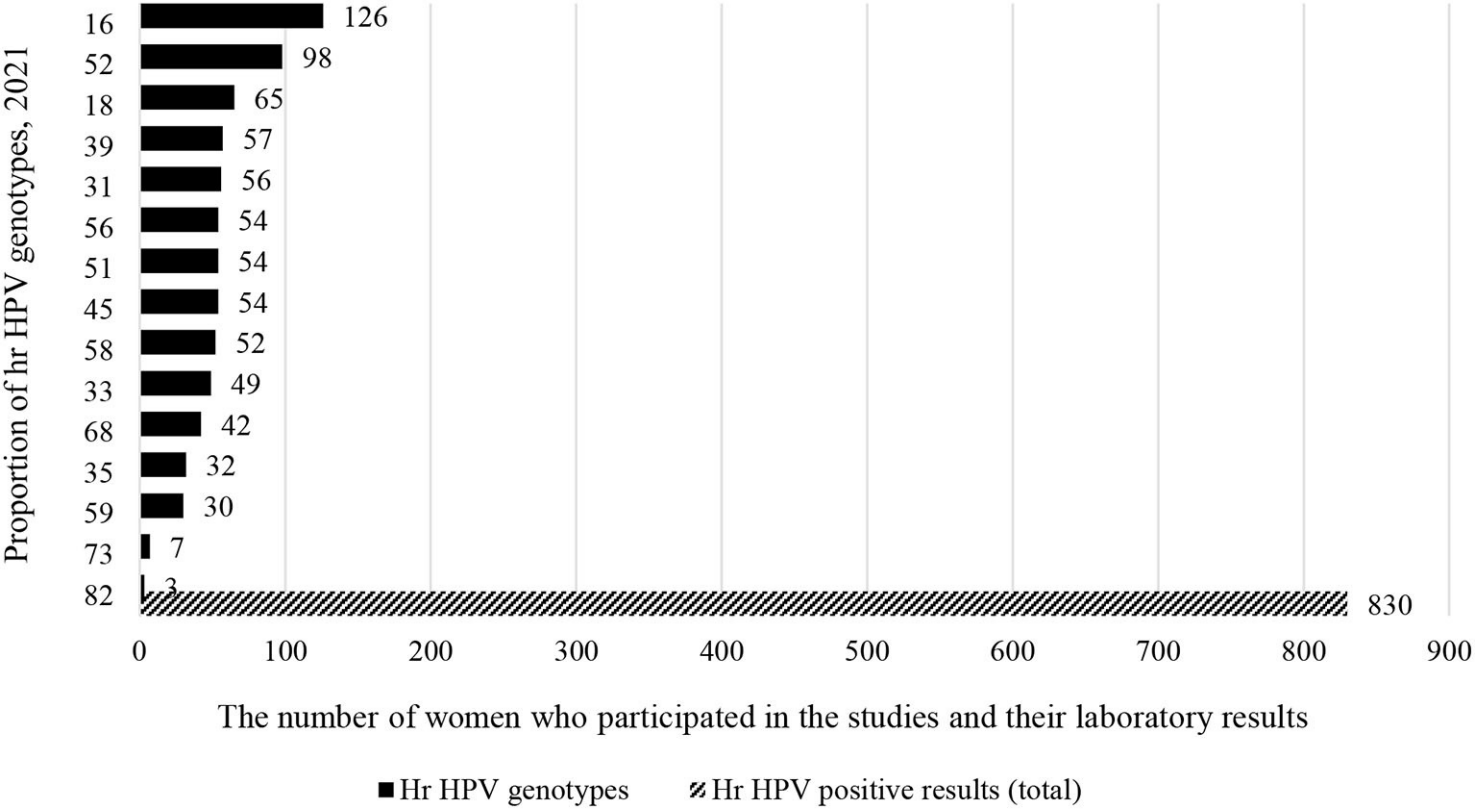
Type-specific sub-group prevalence of hr HPV in East Africa, 2021.

Studies conducted in Ethiopia (3), Tanzania (2), Kenya (2), Uganda (2), and Botswana (1) were considered for meta-analysis to determine the type-specific sub-group prevalence of hr HPV in East Africa. Accordingly, 830 (27%) out of 3,075 women who were infected with hr HPV. Moreover, more than a quarter (224) of these 830 women are infected with HPV-16 and HPV-52 (Figure 6).

**Figure 7:**
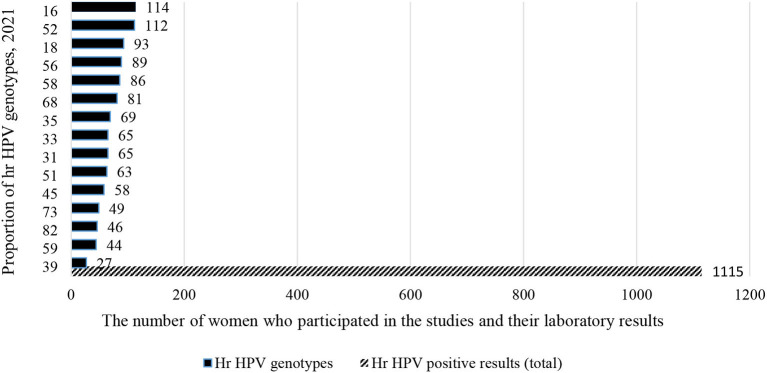
Type-specific sub-group prevalence of hr HPV in Southern African countries, 2021.

**Figure 8:**
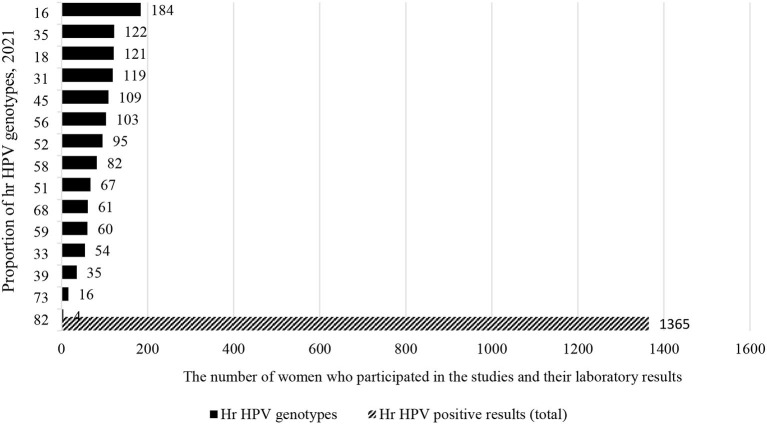
Type-specific sub-group prevalence of hr HPV in West Africa, 2021.

In the meantime, a total of 1,115 (36.2%) out of 3,075 women were from Southern African countries. As seen in the eastern part of Africa, similarly, studies show that about one-fifth [226 (20.3%)] of women infected with HPV-16 and HPV-52 (Figure 7). In contrary to the above two sub-group prevalence, in the Western African countries, the genotype distribution of hr HPV is different, and more than one-fifth of the women surveyed [306/1,365 (22.4%)] were infected with HPV-16 and HPV-35 (Figure 8).

**Figure 9:**
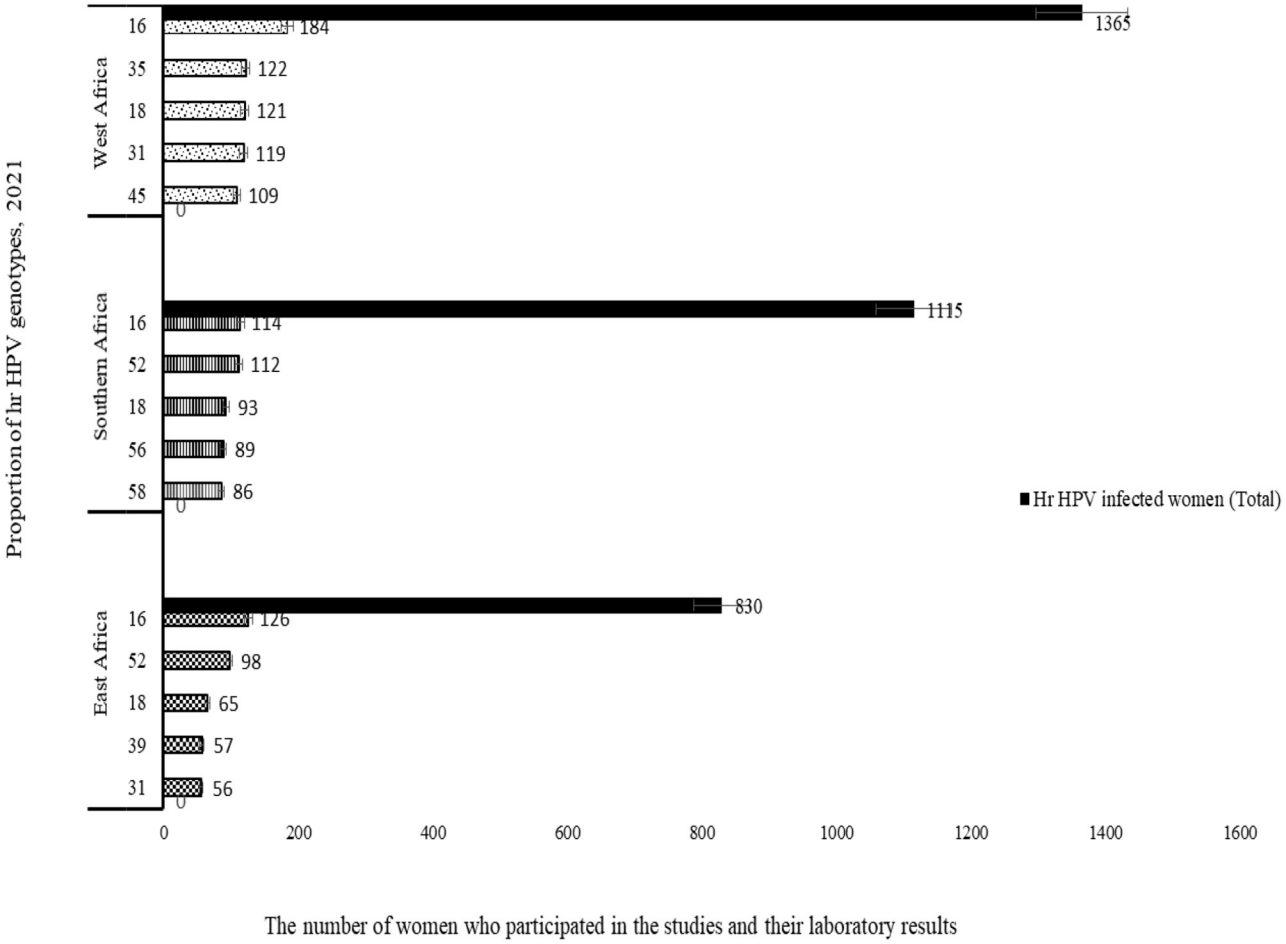
A summary of type-specific sub-group prevalence of hr HPV in sub-Saharan Africa, 2021.

In general, the distribution of hr HPV genotypes from one sub-group to another is inconsistent with the exception of the primary and tertiary genotypes of the virus (Figure 9). Therefore, it is clear from this analysis that a study about the prevalence and mapping the genotype distribution of hr HPV in each country are very crucial to implement an effective vaccine-based prevention strategy.

## Discussion

Getting up-to-date and organized information about the pooled and type-specific prevalence of hr HPV helps to identify the underlying burden of the disease, evaluate the role of HPV vaccines in the prevention of the disease, and develop home-based diagnostic tests. With all these points in mind, this systematic review and meta-analysis have calculated the representative estimations of the type-specific prevalence and genotype distribution of hr HPV in sub-Saharan African countries.

According to this systematic review, the prevalence of hr HPV varies from 10.7% (8) to 97.2% (10) for various reasons. For example, the health status of the women we studied was one of the main reasons. This means that we find it lower in healthy women with low-grade precancerous lesions, and that the prevalence is higher in WLWH with weakened immune systems. Therefore, the results of this systematic review tell us that when we determine the prevalence of hr HPV infection and when we develop strategies for the prevention and treatment of the disease, we should take into account the health and local/ social context of the program's clients.

The pooled prevalence of hr HPV in the sub-Saharan African countries is 32.3% which is lower than the previously reported 50.5% pooled prevalence in 2015 (40). In a review paper published by Ogembo et al. the inclusion of a large number of women who have pre-cancerous lesions in the study might be a factor for the high pooled prevalence of the disease. In this case, it is difficult to know the actual estimation for the pooled prevalence of the disease, so it is important to proportionally take different stages of the disease.

The type-specific prevalence of hr HPV among the sub-groups of sub-Saharan African countries was not uniformly distributed. According to this systematic review and meta-analysis, HPV genotypes HPV-16,−52,−18,−39, and−31 are widely distributed in the eastern part of Africa. When we look at studies to ensure the consistency of the genotype distribution, for example, in a study in Ethiopia, the most prevalent genotypes were hr-HPV- 16,−52,−18,−58, and−45 (41), which is somewhat similar to the results of this study. In Kenya, one study has revealed that the hr HPV-52,−51,−16,−35 and−18 were the most prevalent genotypes in the first months of pregnancy. But in the last months of pregnancy, 7 cases had a persistent HPV infection as a result of HPV-16 and−18 (42). The genotype distribution was quite different from the early stage of pregnancy.

This meta-analysis has revealed that, in the Southern African region, the pooled distribution of HPV-16,−52,−18,−56, and−58 genotypes have been slightly different from that of East Africa. However, at the country level, in a study involving high school students in South Africa, the genotype distribution was significantly different. HPV-58 was the most commonly detected type, followed by HPV-52,−45,−16, and−18 (43). Similarly, in Mozambique HPV-52 is the most common genotype, followed by HPV-35 and−16. This distribution is very similar to the results of a study in South Africa (44). This showed us the distribution of genotypes at the country level may be different from the distribution at the sub-continental level. Therefore, each country needs to identify the distribution of genotypes at the national level before embarking on immunization-based disease prevention strategy.

On the other hand, the pooled HPV genotype distribution in the West African countries has been also characterized in a different form from the East and southern African sub- regions. That is, HPV-16,−35,−18,−31, and−45. In Benin, among Female Sexual Workers (FSWs) the three most common genotypes are HPV-58,−16, and−52. Thus, we have seen that the distribution of HPV genotypes differs between key vulnerable populations and the general population (35).

## Limitations

The study had the following limitations. The study reviewed and analyzed studies conducted using an observational study design i.e., cross-sectional study design. This type of study design is less likely to predict causal-effect correlation. As a result, we are often exposed to bias, which leads to wrong conclusions. Authors were reviewed and realized that studies included in the review have highly variable sample size. This might potentially leads to interfere the nature of the study's representativeness. It also included studies that were conducted using different diagnostic tools. This may cause differences in sensitivity and specificity.

## Conclusion and Recommendations

HPV is currently increasing in sub-Saharan Africa countries and it is becoming a major public health problem. Similarly, the distribution of genotypes is inconsistent at the continental level and even varies from place to place within a country.

HPV infection has a known and effective vaccine. But the production of the vaccine and its effectiveness will be measured when a vaccination program based on the type of virus is launched in a country. Thus, taking into account the key factors mentioned above, each country should have its own information on the prevalence of HPV infection and the type of virus. Doing so will help to develop and implement an effective vaccine-based disease prevention program.

## Author Contributions

Conceptualization: AS. Data curation and formal analysis: AS and NA. Investigation, methodology, and validation: AS, NA, TG, BS, AnM, and AdM. Software and writing—original draft: AS. Supervision and writing—review and editing: NA, TG, BS, AnM, and AdM. All authors contributed to the article and approved the submitted version.

## Conflict of Interest

The authors declare that the research was conducted in the absence of any commercial or financial relationships that could be construed as a potential conflict of interest.

## Publisher's Note

All claims expressed in this article are solely those of the authors and do not necessarily represent those of their affiliated organizations, or those of the publisher, the editors and the reviewers. Any product that may be evaluated in this article, or claim that may be made by its manufacturer, is not guaranteed or endorsed by the publisher.
