# Supplementary material for: Prevalence and Genotype Distribution of High-Risk Human Papillomavirus Infection Among Sub-Saharan African Women: A Systematic Review and Meta-Analysis
Source: Front Public Health. 2022 Jul 8;10:890880. doi: 10.3389/fpubh.2022.890880 (PMC9304908; doi:10.3389/fpubh.2022.890880)
Supplement: Supplementary file 1 [file Data_Sheet_1.PDF]

Appendix S1: Detailed search strategy used to request the electronic databases (PubMed/ MEDLINE)

1,730 records returned from August 1 to September 15, 2021

(Papillomavirus [Mesh] OR Papillomavirus Vaccines [Mesh] OR papilloma* [tiab] OR HPV* [tiab]) AND (Cervical [tiab] OR cervix [tiab] OR uter*[tiab])

AND

(Uterine Cervical Dysplasia [Mesh] OR Uterine Cervical Neoplasms[Mesh] OR HSIL*[tiab] OR high grade squamous intraepithelial[tiab] OR cancer*[tiab] OR tumor[tiab] OR tumors[tiab] OR tumoral*[tiab] OR neoplas*[tiab] OR tumour*[tiab] OR dysplasia*[tiab] OR dysplastic[tiab] OR carcino*[tiab] OR adenosquam*[tiab] OR adenocarcinoma*[tiab])

AND

("Sub Saharan Africa "[Mesh] OR Africa [all fields] OR Southern Africa [all fields] OR West Africa[all fields] OR Western Africa[all fields] OR Central Africa[all fields] OR West African[all fields] OR East African[all fields] OR South African[tiab] OR Central African[tiab] OR Mauritania [all fields] OR Senegal [all fields] OR Gambia [all fields] OR Guinea-bissau [all fields] OR Mali [all fields] OR Guinea [all fields] OR Sierra leone [all fields] OR Liberia [all fields] OR Cote d'ivoire [all fields] OR Nigeria [all fields] OR Niger [all fields] OR Chad [all fields] OR Burkina faso [all fields] OR Ghana [all fields] OR Togo [all fields] OR Benin [all fields] OR Equatorial Guinea [all fields] OR Sao Tome and Principe [all fields] OR Congo [all fields] OR Rwanda [all fields] OR Burundi [all fields] OR Uganda [all fields] OR Djibouti [all fields] OR Seychelles [all fields] OR Comoros[all fields] OR Mauritius [all fields] OR Malawi [all fields] OR Swaziland [all fields] OR Lesotho [all fields] OR Central African Republic [all fields] OR Cameroon [all fields] OR Gabon [all fields] OR Sudan [all fields] OR South Sudan [all fields] OR Eritrea [all fields] OR Ethiopia [all fields] OR DRC [all fields] OR Democratic Republic of Congo [all fields] OR Kenya [all fields] OR Somalia [all fields] OR Tanzania [all fields] OR Angola [all fields] OR Zambia [all fields] OR Namibia [all fields] OR Zimbabwe [all fields] OR Botswana [all fields] OR Mozambique [all fields] OR Madagascar [all fields] OR South Africa[all fields]
