# Supplementary material for: Prevalence and Genotype Distribution of High-Risk Human Papillomavirus Infection Among Sub-Saharan African Women: A Systematic Review and Meta-Analysis
Source: Front Public Health. 2022 Jul 8;10:890880. doi: 10.3389/fpubh.2022.890880 (PMC9304908; doi:10.3389/fpubh.2022.890880)
Supplement: Supplementary file 2 [file Data_Sheet_2.PDF]

Table S1- The Preferred Reporting Items for Systematic Review and Meta-Analysis Protocols (PRISMA-P) checklist in 2015

Section/topic	#	Checklist item	Reported on page #
**TITLE**			
Title	1	Identify the report as a systematic review, meta-analysis, or both.	
**ABSTRACT**			
Structured summary	2	Provide a structured summary including, as applicable: background; objectives; data sources; study eligibility criteria, participants, and interventions; study appraisal and synthesis methods; results; limitations; conclusions and implications of key findings; systematic review registration number.	
**INTRODUCTION**		***There is no comparison because it's a prevalence study**	
Rationale	3	Describe the rationale for the review in the context of what is already known.	
Objectives	4	Provide an explicit statement of questions being addressed with reference to participants, interventions, comparisons, outcomes, and study design (PICOS).	
**METHODS**			
Protocol and registration	5	Indicate if a review protocol exists, if and where it can be accessed (e.g., Web address), and, if available, provide registration information including registration number.	
Eligibility criteria	6	Specify study characteristics (e.g., PICOS, length of follow-up) and report characteristics (e.g., years considered, language, publication status) used as criteria for eligibility, giving rationale.	
Information sources	7	Describe all information sources (e.g., databases with dates of coverage, contact with study authors to identify additional studies) in the search and date last searched.	
Search	8	Present full electronic search strategy for at least one database, including any limits used, such that it could be repeated.	
Study selection	9	State the process for selecting studies (i.e., screening, eligibility, included in systematic review, and, if applicable, included in the meta-analysis).	

Section/topic	#	Checklist item	Reported on page #
Data collection process	10	Describe method of data extraction from reports (e.g., piloted forms, independently, in duplicate) and any processes for obtaining and confirming data from investigators.	
Data items	11	List and define all variables for which data were sought (e.g., PICOS, funding sources) and any assumptions and simplifications made.	
Risk of bias in individual studies	12	Describe methods used for assessing risk of bias of individual studies (including specification of whether this was done at the study or outcome level), and how this information is to be used in any data synthesis.	
Summary measures	13	State the principal summary measures (e.g., risk ratio, difference in means).	
Synthesis of results	14	Describe the methods of handling data and combining results of studies, if done, including measures of consistency (e.g., I^2) for each meta-analysis.	
Risk of bias across studies	15	Specify any assessment of risk of bias that may affect the cumulative evidence (e.g., publication bias, selective reporting within studies).	
Additional analyses	16	Describe methods of additional analyses (e.g., sensitivity or subgroup analyses, meta-regression), if done, indicating which were pre-specified.	
**RESULTS**			
Study selection	17	Give numbers of studies screened, assessed for eligibility, and included in the review, with reasons for exclusions at each stage, ideally with a flow diagram.	
Study characteristics	18	For each study, present characteristics for which data were extracted (e.g., study size, PICOS, follow-up period) and provide the citations.	
Risk of bias within studies	19	Present data on risk of bias of each study and, if available, any outcome-level assessment (see Item 12).	
Results of individual studies	20	For all outcomes considered (benefits or harms), present, for each study: (a) simple summary data for each intervention group and (b) effect estimates and confidence intervals, ideally with a forest plot.	
Synthesis of results	21	Present results of each meta-analysis done, including confidence intervals and measures of consistency.	

Section/topic	#	Checklist item	Reported on page #
Risk of bias across studies	22	Present results of any assessment of risk of bias across studies (see Item XX).	
Additional analysis	23	Give results of additional analyses, if done (e.g., sensitivity or subgroup analyses, meta-regression).	
**DISCUSSION**			
Summary of evidence	24	Summarize the main findings including the strength of evidence for each main outcome; consider their relevance to key groups (e.g., health care providers, users, and policy makers).	
Limitations	25	Discuss limitations at study and outcome level (e.g., risk of bias), and at review level (e.g., incomplete retrieval of identified research, reporting bias).	
Conclusions	26	Provide a general interpretation of the results in the context of other evidence, and implications for future research.	
**FUNDING**			
Funding	27	Describe sources of funding for the systematic review and other support (e.g., supply of data); role of funders for the systematic review.	

Table S2- Quality Assessment Tool for Observational Cohort and Cross-Sectional Studies

Criteria		Yes	No	Other (CD, NR, NA)*
1.	Was the research question or objective in this paper clearly stated?			
2.	Was the study population clearly specified and defined?			
3.	Was the participation rate of eligible persons at least 50%?			
4.	Were all the subjects selected or recruited from the same or similar populations (including the same time period)? Were inclusion and exclusion criteria for being in the study pre-specified and applied uniformly to all participants?			
5.	Was a sample size justification, power description, or variance and effect estimates provided?			
6.	For the analyses in this paper, were the exposure(s) of interest measured prior to the outcome(s) being measured?			
7.	Was the timeframe sufficient so that one could reasonably expect to see an association between exposure and outcome if it existed?			
8.	For exposures that can vary in amount or level, did the study examine different levels of the exposure as related to the outcome (e.g., categories of exposure, or exposure measured as continuous variable)?			
9.	Were the exposure measures (independent variables) clearly defined, valid, reliable, and implemented consistently across all study participants?			
10.	Was the exposure(s) assessed more than once over time?			
11.	Were the outcome measures (dependent variables) clearly defined, valid, reliable, and implemented consistently across all study participants?			
12.	Were the outcome assessors blinded to the exposure status of participants?			
13.	Was loss to follow-up after baseline 20% or less?			
14.	Were key potential confounding variables measured and adjusted statistically for their impact on the relationship between exposure(s) and outcome(s)?			

*CD, cannot determine; NA, not applicable; NR, not reported

Table S3. Key information retrieved for systematic review and meta-analysis

S.No	Study name and year of publication	Regional category	Country	Diagnostic method	Specimen	Study design	Event	Sample size	Prevalence (95% CI)
1.	Teka, B., et al. (2021)	E.A	Ethiopia	PCR	Cervical swab	CS	157	764	20.5% (20.79-20.21)
2.	Ali, K. E., et al. (2019)	E.A	Ethiopia	PCR	Cervical swab	CS	50	366	13.7% (10.50-17.60)
3.	Asiimwe, S., et al., (2008)	E.A	Uganda	Hybrid capture 2	Genital swabs	CS	54	314	17.2% (13.4 – 21.8)
4.	Blossom D.B., et al., (2007)	E.A	Uganda	PCR	Cervical & vaginal swab	CS	49	106	46.2% (37.0 – 55.7)
5.	Dartell M., et al., (2012)	E.A	Tanzania	Hybrid capture 2	Cervical swab	CS	725	3,603	20.1% (18.8 – 21.5)
6.	De Vuyst H.,et al., (2010)	E.A	Kenya	PCR	Cervical swab	CS	208	496	41.9% (37.7 – 46.3)
7.	Leyh-Bannurah S., et al., (2014)	E.A	Ethiopia	Hybrid capture 2	Cervical swab	CS	94	537	17.5% (14.5 – 21.0)
8.	Mayaud P., et al., (2003)	E.A	Tanzania	PCR	Cervical and vaginal swab	CS	190	561	33.9% (30.1 – 37.9)
9.	Rahman M., et al., (2011)	E.A	Kenya	DNA microarray	Cervical swab	CS	240	488	49.2% (44.8 – 53.6)
10.	Castle, P. E., et al. (2020)	E.A	Ethiopia	PCR	Cervical swab	CS	257	1,022	25.2% (21.2–29.4%)
11.	Rosa Catarino, et al., (2015)	S.A	Madagascar	Real time PCR	Cervical swab	CS	424	1081	39.2% (36.4 – 42.2)
12.	Dols, J ,et al., (2012)	S.A	South Africa	PCR	Cervical swab	CS	215	258	83.3% (78.3 – 87.4)
13.	Gravitt P., et al., (2012)	S.A	Zimbabwe	PCR	Cervical swab	CS	226	423	53.4% (48.7 – 58.1)
14.	Sahasrabuddhe V., et al., (2007)	S.A	Zambia	PCR	Cervical swab	CS	141	145	97.2% (92.9 – 99.0)
15.	Tayib S., et al., (2015)	S.A	South Africa	Hybrid capture 2	Cervical swab	CS	106	156	67.9% (60.2 – 74.8)
16.	Zoa Assoumou., et al. (2016)	W.A	Gabon	Nested-PCR	Cervical swab	CS	120	200	60.0% (53.1 – 66.6)
17.	Domfeh A., et al., (2008)	W.A	Ghana	PCR	Cervical swab	CS	8	75	10.7% (5.4 – 19.9)
18.	Jaquet A., et al., (2012)	W.A	Cote d' Ivorie	PCR	Cervical swab	CS	463	510	90.8% (87.9 – 93.0)
19.	Kunckler M., et al., (2017)	W.A	Cameroon	Xpert HPV assay	Virginal swab	CS	187	1012	18.5% (16.2 – 21.0)
20.	Manga M., et al., (2015)	W.A	Nigeria	PCR	Cervical swab	CS	100	208	48.1% (41.4 – 54.9)
21.	Mbaye el., et al., (2014)	W.A	Senegal	PCR	Cervical swab	CS	214	936	22.9% (20.3 – 25.7)
22.	Obiri-Yeboah D., et al (2017)	W.A	Ghana	Hybrid capture 2	Cervical swab	CS	192	329	58.4%(53.0 – 63.6)
23.	Okolo C., et al., (2010)	W.A	Nigeria	PCR	Cervical swab	CS	245	932	26.3% (23.6 – 29.2)
24.	Ouedraogo C., et al., (2011)	W.A	Burkina Faso	PCR	Cervical swab	CS	230	256	90.0% (85.0 – 93.0)
25.	Piras F., et al., (2011)	W.A	Benin	PCR	Cervical swab	CS	142	427	33.3% (28.9 – 37.9)
26.	Wall S., et al., (2005)	W.A	Gambia	PCR	Cervical swab	CS	138	1,061	13.0% (11.1 – 15.2)
27.	Kuassi-Kpede, A. P., et al. (2021)	WA	Togo	Real time PCR	Cervical swab	CS	128	240	53.3% (47 - 59.6)

C.A= Central African E.A= Eastern African S.A= Southern African W.A= Western African PCR= Polymerase chain reaction CS= Cross sectional CI= Confidence Interval

Table S4. Overall genotyping of HR-HPV among sub Saharan African countries, 2021

Regional category	First author name	HR-HPV 16			HR-HPV 18			HR-HPV 31			HR-HPV 33			HR-HPV 35		
		Event	Sample size	Event rate	Event	Sample size	Event rate	Event	Sample size	Event rate	Event	Sample size	Event rate	Event	Sample size	Event rate
EA	De Vuyst H. (Kenya) , 2010	38	208	0.18	23	208	0.11	9	208	0.04	15	208	0.03	3	208	0.01
EA	Leyh-Bannurah S (Ethiopia), 2014	21	86	0. 24	3	86	0.04	9	86	0.10	2	86	0.02	5	86	0.06
EA	Mayaud P. (Tanzania), 2003	34	190	0.18	13	190	0.07	8	190	0.04	20	190	0.10	3	190	0.02
EA	Rahman M(Kenya), 2011	25	240	0.10	17	240	0.07	30	240	0.12	12	240	0.05	21	240	0.09
EA	Blossom D (Uganda), 2007	8	106	0.07	9	106	0.08	**0**	106	0.00	0	106	0.00	0	106	0.00
		**126**	**830**		**65**	**830**		**56**	**830**		**49**	**830**		**32**	**830**	
SA	Catarino R (Madagascar), 2016	32	586	0.06	32	586	0.06	35	586	0.06	25	586	0.04	46	586	0.08
SA	Tayib S(South Africa), 2015	8	106	0.08	8	106	0.08	2	106	0.02	2	106	0.02	1	106	0.01
SA	Gravitt P.(Zimbabwe), 2012	74	423	0.17	53	423	0.12	28	423	0.07	38	423	0.09	22	423	0.05
		**114**	**1115**		**93**	**1115**		**65**	**1115**		**65**	**1115**		**69**	**1115**	
WA	Kunckler M (Cameroon), 2017	18	187	0.10	29	187	0.16	0	187	0.00	2	187	0.01	2	187	0.01
WA	Mbaye el H(Senegal), 2014	22	214	0.10	13	214	0.06	44	214	0.21	13	214	0.06	6	214	0.03
WA	Obiri-Yeboah D. (Ghana), 2017	18	329	0.06	20	329	0.06	14	329	0.04	15	329	0.05	28	329	0.08
WA	Okolo C (Nigeria), 2010	30	245	0.12	18	245	0.07	27	245	0.11	5	245	0.02	30	245	0.12
WA	Piras F(Benin), 2011	25	142	0.18	21	142	0.15	5	142	0.03	6	142	0.04	32	142	0.22
WA	Zoa Assoumou S (Gabon), 2016	68	120	0.57	2	120	0.02	4	120	0.03	10	120	0.08	2	120	0.02
WA	Kuassi-Kpede, A. P., et al. (Togo), 2011	3	128	0.02	18	128	0.14	25	128	0.19	3	128	0.02	22	128	0.17
		**184**	**1365**		**121**	**1365**		**119**	**1365**		**54**	**1365**		**122**	**1365**	

EA- Eastern Africa SA- Southern Africa WA- Western Africa

Regional category	First author name	HR-HPV 39			HR-HPV 45			HR-HPV 51			HR-HPV 52			HR-HPV 56		
		Event	Sample size	Event rate	Event	Sample size	Event rate	Event	Sample size	Event rate	Event	Sample size	Event rate	Event	Sample size	Event rate
EA	De Vuyst H. (Kenya) , 2010	5	208	0.02	11	208	0.05	16	208	0.08	20	208	0.10	8	208	0.04
EA	Leyh-Bannurah S (Ethiopia), 2014	5	86	0.06	4	86	0.05	6	86	0.07	10	86	0.12	9	86	0.11
EA	Mayaud P. (Tanzania), 2003	1	190	0.01	1	190	0.01	7	190	0.04	2	190	0.01	2	190	0.01
EA	Rahman M(Kenya), 2011	46	240	0.19	38	240	0.16	25	240	0.10	51	240	0.21	35	240	0.15
EA	Blossom D (Uganda), 2007	0	106	0.00	0	106	0.00	**0**	106	0.00	15	106	0.14	0	106	0.00
		**57**	**830**		**54**	**830**		**54**	**830**		**98**	**830**		**54**	**830**	
SA	Catarino R (Madagascar), 2016	8	586	0.01	25	586	0.04	23	586	0.04	53	586	0.09	29	586	0.05
SA	Tayib S(South Africa), 2015	4	106	0.04	8	106	0.07	5	106	0.05	5	106	0.05	35	106	0.33
SA	Gravitt P.(Zimbabwe), 2012	15	423	0.03	25	423	0.06	35	423	0.08	54	423	0.13	25	423	0.06
		**27**	**1115**		**58**	**1115**		**63**	**1115**		**112**	**1115**		**89**	**1115**	
WA	Kunckler M (Cameroon), 2017	2	187	0.01	29	187	0.15	2	187	0.01	2	187	0.01	2	187	0.01
WA	Mbaye el H(Senegal), 2014	9	214	0.04	23	214	0.10	18	214	0.08	33	214	0.15	15	214	0.02
WA	Obiri-Yeboah D. (Ghana), 2017	10	329	0.03	12	329	0.04	2	329	0.01	19	329	0.06	19	329	0.06
WA	Okolo C (Nigeria), 2010	6	245	0.02	19	245	0.08	14	245	0.06	13	245	0.05	22	245	0.09
WA	Piras F(Benin), 2011	1	142	0.01	14	142	0.10	5	142	0.04	4	142	0.03	12	142	0.09
WA	Zoa Assoumou S (Gabon), 2016	0	120	0.00	2	120	0.02	0	120	0.00	0	120	0.00	4	120	0.03
WA	Kuassi-Kpede, A. P., et al. (Togo), 2011	7	128	0.06	10	128	0.08	26	128	0.20	24	128	0.19	29	128	0.23
		**35**	**1365**		**109**	**1365**		**67**	**1365**		**95**	**1365**		**103**	**1365**	

EA- Eastern Africa SA- Southern Africa WA- Western Africa

Regional category	First author name	HR-HPV 58			HR-HPV 59			HR-HPV 68			HR-HPV 73			HR-HPV 82		
		Event	Sample size	Event rate	Event	Sample size	Event rate	Event	Sample size	Event rate	Event	Sample size	Event rate	Event	Sample size	Event rate
EA	De Vuyst H. (Kenya) , 2010	8	208	0.04	6	208	0.03	8	208	0.04	6	208	0.03	2	208	0.01
EA	Leyh-Bannurah S (Ethiopia), 2014	2	86	0.02	1	86	0.01	4	86	0.05	1	86	0.01	1	86	0.01
EA	Mayaud P. (Tanzania), 2003	23	190	0.12	4	190	0.02	5	190	0.03	0	190	0.00	0	190	0.00
EA	Rahman M(Kenya), 2011	11	240	0.05	19	240	0.08	25	240	0.10	0	240	0.00	0	240	0.00
EA	Blossom D (Uganda), 2007	8	106	0.06	0	106	0.00	**0**	106	0.00	0	106	0.00	0	106	0.00
		52	830		30	830		**42**	830		7	830		3	830	
SA	Catarino R (Madagascar), 2016	27	586	0.05	28	586	0.05	59	586	0.10	35	586	0.06	22	586	0.04
SA	Tayib S(South Africa), 2015	5	106	0.05	5	106	0.05	4	106	0.04	3	106	0.03	1	106	0.01
SA	Gravitt P.(Zimbabwe), 2012	54	423	0.13	11	423	0.03	18	423	0.04	11	423	0.03	23	423	0.05
		**86**	**1115**		**44**	**1115**		**81**	**1115**		**49**	**1115**		**46**	**1115**	
WA	Kunckler M (Cameroon), 2017	2	187	0.01	2	187	0.01	2	187	0.01	2	187	0.01	0	187	0.00
WA	Mbaye el H(Senegal), 2014	3	214	0.01	6	214	0.01	28	214	0.03	3	214	0.01	0	214	0.00
WA	Obiri-Yeboah D. (Ghana), 2017	25	329	0.08	10	329	0.03	18	329	0.06	0	329	0.00	0	329	0.00
WA	Okolo C (Nigeria), 2010	21	245	0.09	0	245	0.00	2	245	0.01	4	245	0.02	4	245	0.02
WA	Piras F(Benin), 2011	19	142	0.13	35	142	0.25	0	142	0.00	7	142	0.05	0	142	0.00
WA	Zoa Assoumou S (Gabon), 2016	0	120	0.00	0	120	0.00	0	120	0.00	0	120	0.00	0	120	0.00
WA	Kuassi-Kpede, A. P., et al. (Togo), 2011	12	128	0.09	7	128	0.06	11	128	0.09	0	128	0.00	0	128	0.00
		**82**	**1365**		**60**	**1365**		**61**	**1365**		**16**	**1365**		**4**	**1365**	

EA- Eastern Africa SA- Southern Africa WA- Western Africa
